# Unilateral Ptosis in a 72-Year-Old Male: Early Diagnosis of Myasthenia Gravis Using the Ice Pack Test

**DOI:** 10.7759/cureus.73581

**Published:** 2024-11-13

**Authors:** Paulo Conceição, Renato Gonçalves, Ana Reinas

**Affiliations:** 1 Internal Medicine, Centro Hospitalar Universitário de Santo António, Porto, PRT; 2 Internal Medicine, Unidade Local de Saúde da Cova da Beira, Porto, PRT

**Keywords:** bedside clinical examination skills, diagnosis, ice pack test, myasthenia gravis (mg), ocular-myasthenia-gravis, ptosis

## Abstract

Ptosis, or eyelid drooping, can be an early indicator of various neurological and muscular disorders, underscoring the need for accurate diagnosis to ensure proper management. Myasthenia gravis (MG), a neuromuscular disorder, may initially present with isolated ocular symptoms, often resulting in diagnostic delays. This report details the case of a 72-year-old male who presented to the emergency department with a two-week history of progressive unilateral ptosis. A neurological examination, paired with the “ice pack test” - a simple, noninvasive bedside technique - supported the diagnosis of MG. The test improves neuromuscular transmission through cold application, temporarily alleviating symptoms. Early diagnosis using accessible techniques like the ice pack test can facilitate timely intervention, enhancing patient outcomes. This case emphasizes the value of such methods in identifying MG in patients with isolated ocular manifestations.

## Introduction

Myasthenia gravis (MG) is an autoimmune disorder affecting the neuromuscular junction, where antibodies target acetylcholine receptors (AChR) on muscle cells, leading to fluctuating muscle weakness. MG frequently impacts the ocular muscles, often manifesting initially as ptosis or diplopia before involving other muscle groups [1]. Although MG can develop at any age, its onset after age 60 has become increasingly common, particularly in men [2]. Symptoms often begin with ocular involvement, which may mimic other neurological or age-related conditions, complicating and delaying diagnosis [3].

Given the importance of timely diagnosis and intervention, simple bedside tests such as the “ice pack test” offer practical benefits. This test leverages cold-induced inhibition of acetylcholinesterase, the enzyme responsible for acetylcholine breakdown [4]. The temporary reduction in acetylcholinesterase activity can briefly enhance neuromuscular transmission, alleviating ptosis in patients with MG. This case report highlights the ice pack test’s utility in diagnosing MG in an elderly patient with isolated ocular symptoms, illustrating how such accessible methods can aid in the early detection and management of MG.

## Case presentation

A 72-year-old man presented to the emergency department with a two-week history of progressive, painless drooping of his left eyelid. He reported no associated symptoms, such as diplopia, limb weakness, or dysphagia, and had no history of autoimmune or neuromuscular disorders. His medical history was otherwise unremarkable, and he was not taking any medications known to cause muscle weakness.

On physical examination, left-sided ptosis was evident, while ocular movements and pupil responses remained normal, effectively ruling out Horner’s syndrome and other cranial nerve involvement. No additional cranial nerve deficits or neuromuscular abnormalities were noted, and limb strength and reflexes were intact. Given the high suspicion of a neuromuscular disorder, particularly MG, the ice pack test was performed as an initial diagnostic measure.

**Figure 1:**
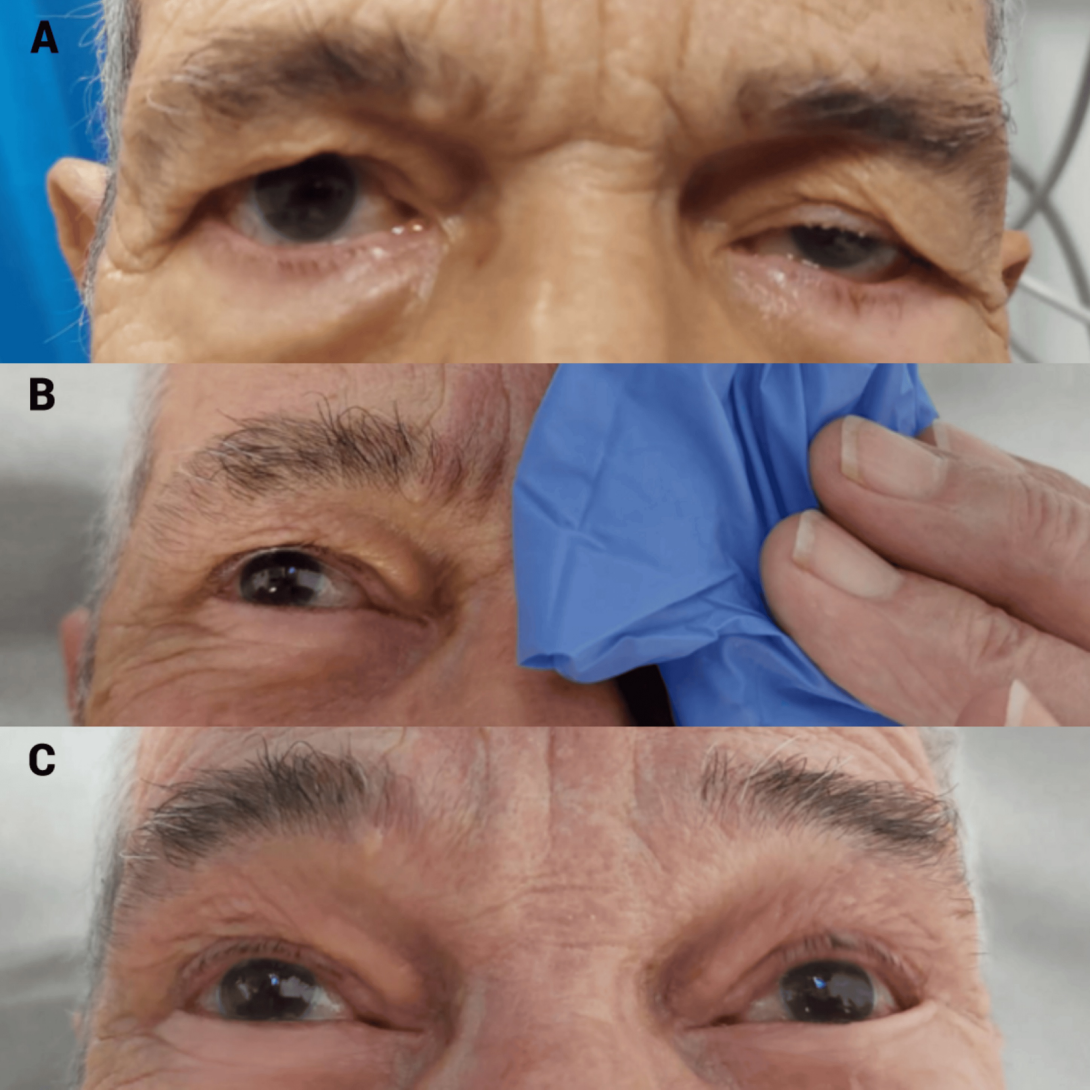
Ice pack application (A) Unilateral left-sided ptosis before intervention. (B) Ice pack applied over the affected left eyelid. (C) Notable improvement in ptosis following ice pack application.

For the test, an ice cube inside a glove was gently applied to the patient’s left eyelid for approximately two minutes. After removal, a notable improvement in the ptosis was observed, strongly suggesting ocular MG (Figure 1). This improvement in ptosis persisted for nearly one minute.

Following the positive ice pack test result, confirmatory tests were conducted, including serum testing for AChR antibodies, which were elevated, and single-fiber electromyography (EMG), which demonstrated characteristic neuromuscular transmission abnormalities associated with MG. The patient was started on pyridostigmine, an acetylcholinesterase inhibitor, resulting in substantial symptomatic improvement.

## Discussion

MG often presents with isolated ocular symptoms, such as ptosis or diplopia, which may lead to misdiagnosis, especially in older adults where other conditions - such as cranial nerve palsy, Horner’s syndrome, or age-related eyelid changes - must also be considered [5]. In elderly patients, distinguishing MG from these conditions is crucial to prevent complications, including the potential progression to generalized muscle weakness if the disease remains undiagnosed [6].

The ice pack test serves as a rapid, noninvasive, and cost-effective tool, particularly useful in such diagnostic scenarios [7]. By temporarily enhancing neuromuscular transmission through cold-induced inhibition of acetylcholinesterase, this bedside test offers a preliminary yet reliable indication of MG, as demonstrated in our patient. Clinically, the ice pack test has shown high sensitivity and specificity, particularly in ocular MG cases [8,9]. Confirmatory testing with AChR antibodies and EMG further validated the diagnosis, enabling timely initiation of treatment.

Early recognition of MG is essential for effective management, as delayed diagnosis can result in significant morbidity, including worsening ocular symptoms or a generalized myasthenic crisis. The ice pack test can help reduce unnecessary diagnostic investigations, allowing for a focused approach when evaluating cases of unilateral ptosis.

Given its clinical efficacy, the ice pack test should be considered a first-line diagnostic approach in assessing unilateral or bilateral ptosis, particularly when symptoms are confined to the ocular region.

## Conclusions

This case highlights the utility of the ice pack test in diagnosing MG early, particularly in patients with isolated ocular symptoms like ptosis. For this 72-year-old patient, the test provided a quick, noninvasive indication of MG, directing further confirmatory testing and facilitating timely treatment. The ice pack test is a practical and cost-effective diagnostic tool that is useful for distinguishing MG from other causes of ptosis in both emergency and outpatient settings. Early diagnosis and intervention can prevent symptom progression and reduce complication risks. Given its simplicity and effectiveness, the ice pack test should be routinely considered for patients presenting with unexplained ptosis, underscoring the role of bedside tests in enhancing diagnostic accuracy and patient outcomes.
